# Jugular foramen tumor causing isolated hypoglossal nerve palsy: a case report

**DOI:** 10.3389/fonc.2026.1635494

**Published:** 2026-01-26

**Authors:** Zhijun Wen, Youcheng Li, Jianhua Cheng, Yirui Huang

**Affiliations:** 1 Department of Neurology, The First Affiliated Hospital of Wenzhou Medical University, Wenzhou, Zhejiang, China; 2 Department of Imaging, The First Affiliated Hospital of Wenzhou Medical University, Wenzhou, Zhejiang, China; 3 Department of Clinical Laboratory, Wenzhou Hospital of Integrated Traditional Chinese and Western Medicine, Wenzhou, Zhejiang, China

**Keywords:** case report, gamma knife, isolated hypoglossal nerve palsy, jugular foramen tumor, schwannoma

## Abstract

**Background:**

The etiology of hypoglossal nerve palsy is diverse, including trauma, infection, tumors, endocrine disorders, autoimmune diseases, and vascular lesions. Tumors in the jugular foramen region are rare and primarily include paragangliomas, schwannomas, or meningiomas. Isolated hypoglossal nerve palsy caused by a jugular foramen tumor is exceedingly rare. This report presents a unique case of a jugular foramen tumor leading to isolated hypoglossal nerve injury.

**Case report:**

A 39-year-old male with no significant medical history presented with a two-week history of headache and 10 days of leftward tongue deviation. Physical examination revealed leftward tongue deviation with mild dysarthria. Nasopharyngoscopy and routine laboratory tests yielded normal results. Lumbar puncture and next-generation sequencing (NGS) ruled out intracranial infection. MRI showed a 6.0×9.0 mm nodule in the left jugular foramen, hyperintense on T2-weighted imaging, isointense on DWI, and exhibiting ring-like enhancement on contrast MRI. A jugular foramen tumor was suspected, and surgical resection was recommended. However, the patient opted for gamma knife treatment at another hospital, resulting in symptom improvement.

**Results and conclusion:**

Multisequence MRI confirmed compression of the hypoglossal nerve by a left jugular foramen tumor. This case is unique in that the tumor, despite its location, manifested solely as hypoglossal nerve palsy, an atypical presentation that can lead to misdiagnosis. This case report highlights the importance of high-resolution imaging and multidisciplinary collaboration in diagnosing skull base lesions and explores the anatomical relationship between the hypoglossal nerve and jugular foramen in disease pathogenesis.

## Introduction

The hypoglossal nerve, a pure motor nerve, rarely presents with isolated injury in clinical practice. Common causes include trauma (1), vascular lesions (2), autoimmune diseases (3), degenerative changes (4), infections (5), and tumor compression (6). The jugular foramen, with its complex anatomy, houses multiple cranial nerves and vascular structures. Although the hypoglossal nerve does not traverse the jugular foramen, its proximity makes it susceptible to compression by nearby lesions (7). Tumors in this region typically affect cranial nerves IX, X, or XI. Reports of jugular foramen tumors presenting solely with hypoglossal nerve symptoms are exceptionally rare. This case, supported by detailed imaging and clinical follow-up, provides valuable insights into the diagnosis and management of such atypical presentations.

## Case presentation

A 39-year-old male presented with a half-a-month history of headache and a 10-day history of dysphagia and leftward tongue deviation. In mid-September 2024, the patient developed persistent, moderate, pressure-like pain in the left craniocervical region, without identifiable precipitating or alleviating factors. He underwent cervical acupuncture therapy at a local hospital for headache relief. While the headache showed slight improvement post-treatment, he developed new-onset dysphagia on the same day, characterized by a sensation of obstruction when swallowing solid foods, along with noticing a leftward deviation of his tongue. Initial evaluation at a local clinic suggested “pharyngitis,” and he was treated with unspecified antibiotics and corticosteroids without symptom improvement. By October 2, 2024, the patient reported worsening dysphagia, accompanied by slurred speech, difficulty chewing, and scalp numbness. He denied any history of odynophagia, fever, or weight loss. There were no other neurological or otological symptoms. For further evaluation, non-contrast brain magnetic resonance imaging (MRI) performed at a local hospital on October 3, 2024, was reported as unremarkable. Seeking a definitive diagnosis, the patient was referred to our outpatient clinic on October 6, 2024. Brain magnetic resonance venography (MRV) revealed an “abnormal signal shadow in the right tongue region,” leading to his admission for further management.

The patient was previously healthy with no significant medical history. He is married, has a 20-pack-year smoking history (approximately one pack per day), and reported a 20-year history of occasional social alcohol consumption. No drug allergies were reported. Family history was negative for similar symptoms, hereditary disorders, or immune-mediated diseases. Notably, the patient came from a middle-class socioeconomic background.

**Figure 1:**
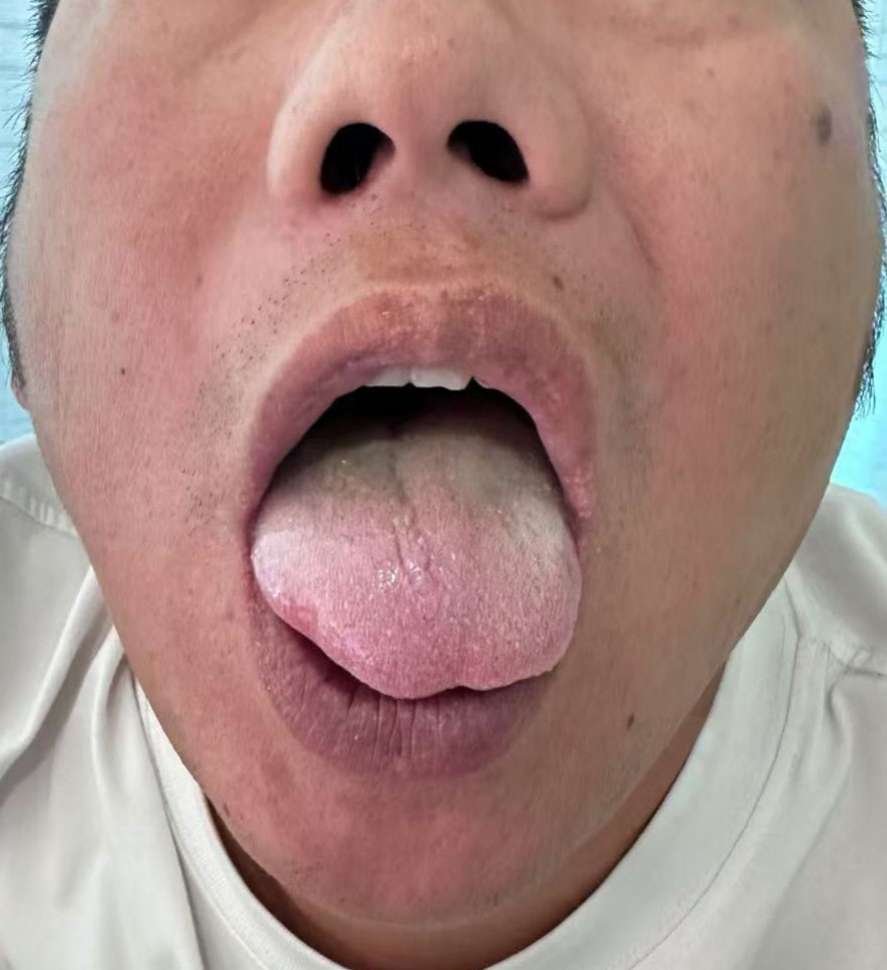
Clinical photograph of the tongue showing leftward deviation upon protrusion, presenting as asymmetry.

On examination, the patient was healthy looking, conscious, alert and oriented to time, place and person. His vitals were unremarkable and he was afebrile. Examination of the nose, throat, paranasal sinuses, and tympanic membranes were normal. No spontaneous nystagmus was noted. Neurological examination revealed mildly slurred speech. Pronounced leftward deviation of the tongue upon protrusion was observed (**Figure 1**). Mild atrophy and spontaneous fasciculations were noted in the left hemi-tongue. The rest of the neurological and systemic examination results were normal.

After he was hospitalized, we performed an analytical study including complete blood cell count, urea, creatinine, electrolytes, uric acid, transaminases, alkaline phosphatase, lactate dehydrogenase, thyroid and parathyroid hormones, calcitonin, C-reactive protein, globular erythrocyte sedimentation rate, rheumatoid factor, complement, anti-nuclear and anti-neutrophil cytoplasmic antibodies, and serology tests for HIV, hepatitis B virus, hepatitis C virus, treponema pallidum, and cytomegalovirus and so on. Also, we completed lumbar puncture and delivered cerebrospinal fluid for Next Generation Sequencing (NGS) examination and pathological examination (Scattered lymphocytes were found in cerebrospinal fluid pathology, but no tumor cells were found), with that, we ruled out intracranial infection.

**Figure 2:**
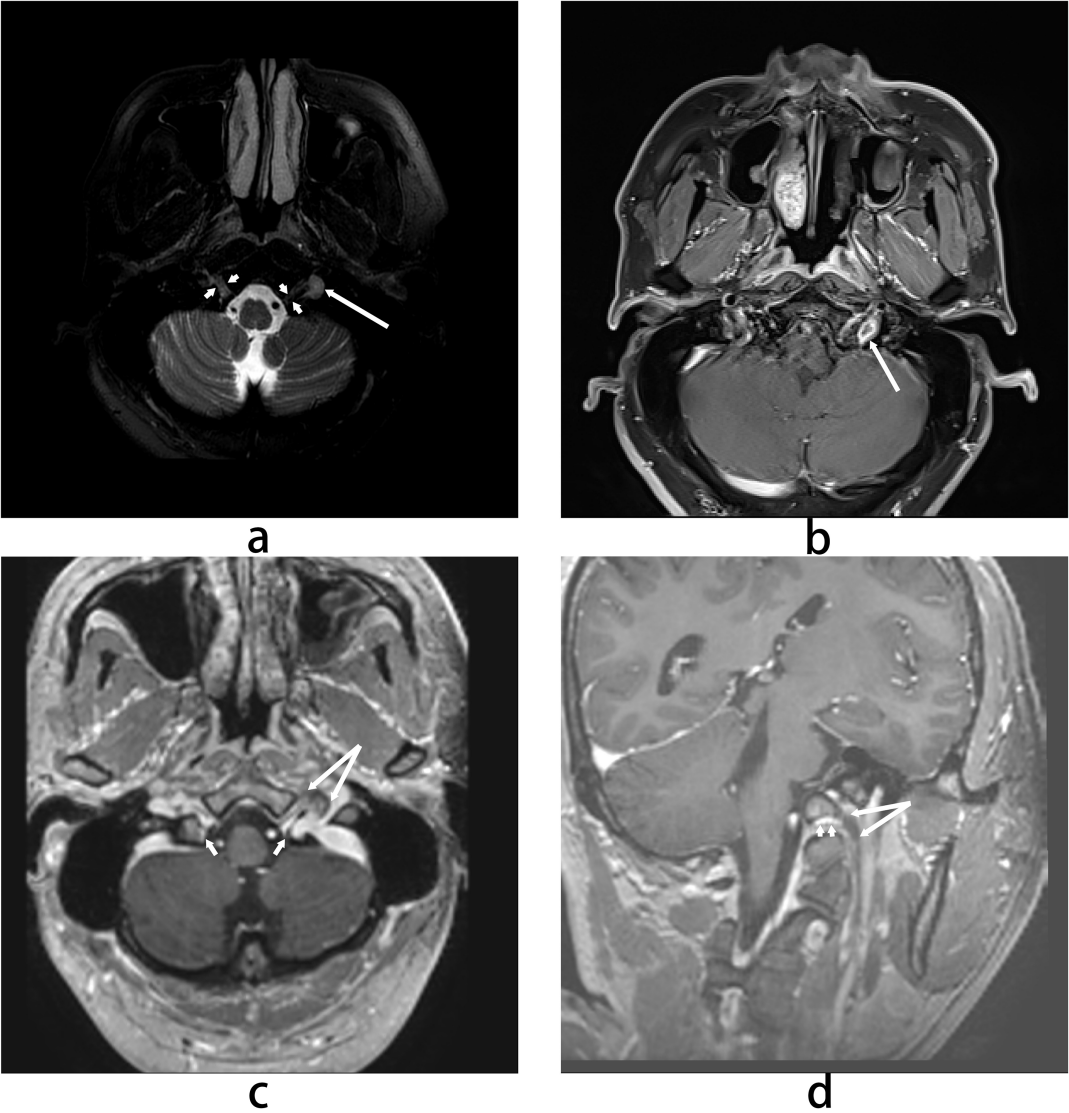
MRI findings. **(a)** Axial T2-weighted image shows a hyperintense nodule (long arrow) adjacent to the hypoglossal nerve (short arrow). **(b)** Axial contrast-enhanced T1-weighted image demonstrates ring-like enhancement of the nodule. **(c)** 3D contrast-enhanced T1-weighted axial reconstruction shows close contact between the extracranial hypoglossal nerve (short arrow) and the tumor, with a thin bony septum (long arrow) between the hypoglossal canal and jugular foramen. **(d)** 3D contrast-enhanced T1-weighted oblique sagittal reconstruction illustrates the intimate relationship between the tumor (long arrow) and the hypoglossal nerve (short arrow).

We also did the multisequence MRI scans, and the results show in **Figure 2**, Combined with multiple examinations of the patient’s head MRV, head MRI and oral MRI, abnormal nodular signal shadow was observed on the left side through the middle of the jugular foramen, with a size of about 6.0x9.0mm, which was hyperintense on T2-WI, isointense on DWI(diffusion weighted imaging), and ring-like enhancement on enhanced MRI, in addition, the unenhanced part of the ring-like enhancing nodule of the left jugular foramina was slightly hyperintense on the ADC(Apparent diffusion coefficient) image(not showed in **Figure 2**), and the curved compression change near the medial margin of the jugular bulb was obvious.

Initial assessment based on the patient’s history, clinical presentation, and imaging findings suggested a preliminary diagnosis of either an inflammatory nodule or a jugular foramen tumor (possibly a schwannoma). The patient was initially treated with a 6-day course of ceftriaxone sodium (2 g, intravenous infusion, once daily) for suspected infection, followed by an 8-day regimen of neurotrophic therapy (vitamin B1–10 mg orally three times daily and mecobalamin 0.5 mg orally three times daily). No significant clinical improvement was observed. After multidisciplinary review, the diagnosis was revised to “left jugular foramen schwannoma, likely involving the hypoglossal nerve.” Surgical resection with biopsy was recommended; however, the patient declined surgery due to concerns about potential nerve injury and was discharged on October 22, 2024, on continued neurotrophic therapy.

**Figure 3:**
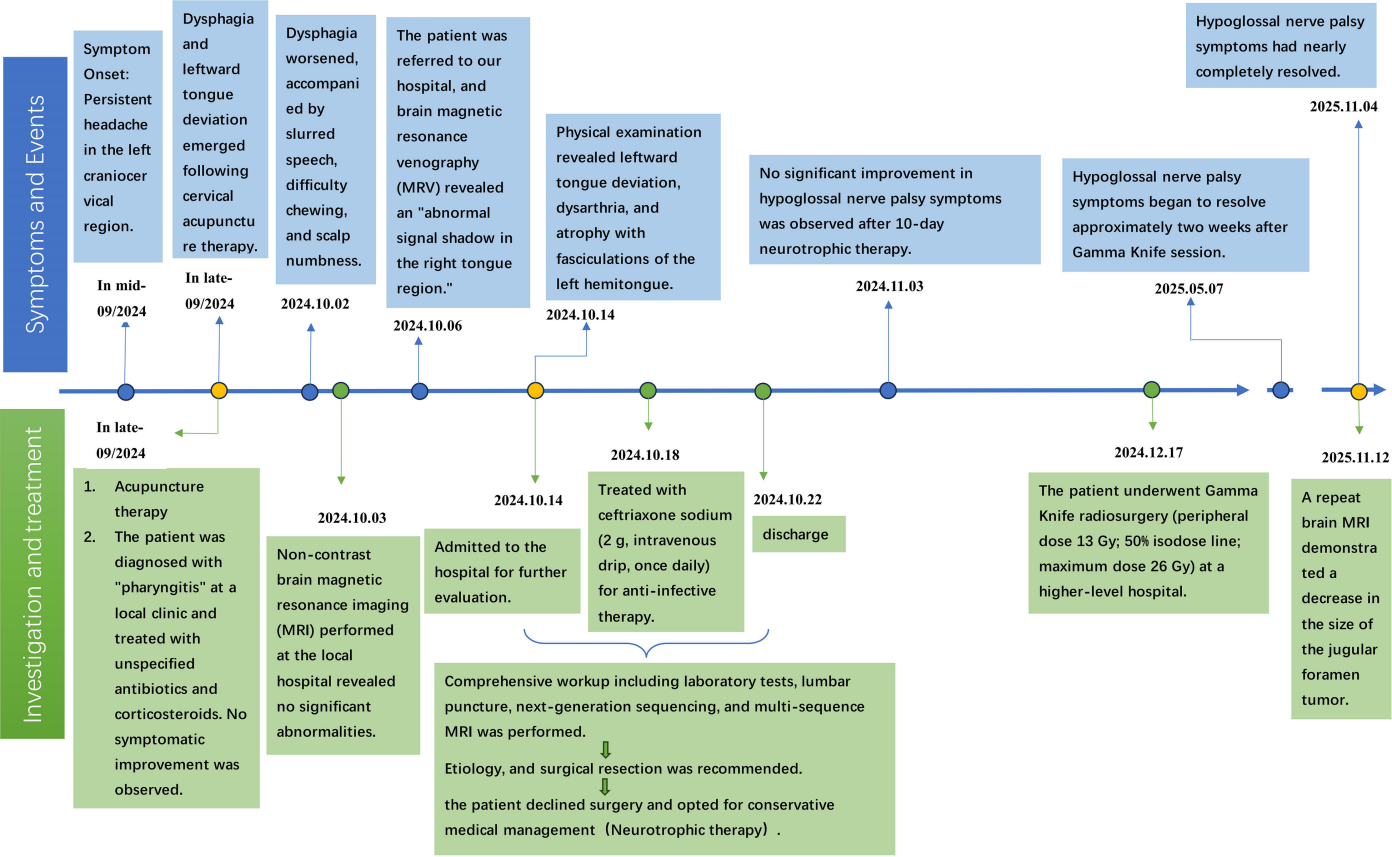
Clinical timeline summarizing key events and interventions.

A follow−up MRI at our institution 10 days post−discharge showed no reduction in tumor size, and symptoms remained unchanged. One month later, telephone follow−up revealed that the patient had been diagnosed with a jugular foramen schwannoma (without pathological confirmation) at another center and had subsequently undergone Gamma Knife radiosurgery with the following parameters: peripheral dose 13 Gy, 50% isodose line, maximum dose 26 Gy. The procedure was uneventful, and the patient reported no acute adverse effects. At the 6−month telephone follow−up, the patient reported having received a single Gamma Knife session, with hypoglossal nerve palsy symptoms beginning to resolve approximately two weeks after treatment. A further follow−up at one year confirmed near−complete resolution of leftward tongue deviation, tongue atrophy, and dysphagia. A repeat brain MRI performed externally on November 12, 2025, demonstrated a decrease in the size of the jugular foramen tumor. The overall clinical timeline is summarized in **Figure 3**.

## Discussion

This case demonstrates an atypical association between a jugular foramen lesion and isolated hypoglossal nerve palsy. Typically, tumors in the jugular foramen region predominantly affect cranial nerves IX, X, or XI. In this case, the exclusive involvement of the hypoglossal nerve may be attributed to a unique anatomical variant: MRI findings were suggestive of an exceptionally thin bony septum between the hypoglossal canal and the jugular foramen, which could have permitted direct compression of the nerve by the tumor.

Although the imaging findings suggested the possibility of a schwannoma, they warrant critical discussion. The lesion exhibited a ring-like enhancement pattern on post contrast T1-weighted images, which is not the classic homogeneous enhancement typically seen in schwannomas. This atypical enhancement pattern raises differential diagnostic considerations, including other jugular foramen pathologies such as paraganglioma, meningioma, or metastatic lesions. Paragangliomas often show intense early enhancement with a “salt and pepper” appearance on T2-weighted images due to flow voids, a feature not prominent in our case. Meningiomas typically present with dural based growth, homogeneous enhancement, and a dural tail sign, none of which were observed here. While the well-defined margins and location of the lesion are consistent with a schwannoma, the absence of pathological confirmation precludes a definitive diagnosis. This imaging ambiguity underscores the importance of considering a broad differential diagnosis for enhancing jugular foramen lesions, even when the clinical presentation suggests nerve compression.

Several diagnostic challenges were encountered. Initially, due to the nonspecific nature of the symptoms, the patient was misdiagnosed with pharyngitis and received inappropriate treatment. The limited resolution of conventional non-contrast MRI also contributed to a false-negative finding in the initial examination. These experiences highlight that for patients with isolated cranial nerve palsy, especially when symptoms show progressive worsening, comprehensive investigations including high resolution MRI should be considered. In this case, the clear delineation of the nerve tumor interface provided by 3D gradient-echo sequences served as the definitive basis for final diagnosis and treatment planning.

Regarding treatment, as indicated by prior research (8), surgical resection remains the preferred approach for large tumors or those causing brainstem compression. We convened a multidisciplinary team for discussion. Given that the tumor had already caused significant neurological deficits with a progressive worsening trend, surgical resection was identified as the optimal treatment strategy. However, skull base surgery carries inherent risks, particularly potential damage to adjacent critical neurovascular structures. After thorough evaluation and detailed physician-patient communication, the patient ultimately opted to undergo Gamma Knife radiosurgery at another hospital.

Studies have demonstrated that Gamma Knife radiosurgery serves as an effective minimally invasive treatment, particularly suitable for small-to-medium sized jugular foramen schwannomas or postoperative residual/recurrent cases, achieving high long term tumor control rates with low complication incidence (9). Therefore, selecting the optimal treatment modality based on tumor characteristics and individual anatomical features is crucial. Compared with surgical resection, Gamma Knife therapy demonstrates superior advantages in preserving cranial nerve function, making it a reasonable alternative choice for this patient. This case highlights the importance of individualized treatment selection based on tumor characteristics, anatomical constraints, and patient preference.

A key limitation of this case is the lack of histopathological verification. The patient declined a biopsy prior to undergoing Gamma Knife therapy. Consequently, the definitive nature of the tumor remains unconfirmed, and the diagnosis of schwannoma is based primarily on clinical and imaging features. This limitation highlights a potential diagnostic dilemma when patients opt for non-surgical interventions without prior tissue sampling. Future management of similar cases should emphasize, where feasible, the value of obtaining a histological diagnosis to guide definitive therapy and prognosis. Another limitation of this study is that the conclusion regarding the presence of an “extremely thin bony septum” between the hypoglossal canal and the jugular foramen was derived from magnetic resonance imaging (MRI) evaluation. However, MRI has inherent limitations in delineating fine bony structures, and its assessment of the thickness and integrity of osseous septa may lack precision. Therefore, confirmation of this anatomical variant requires verification via high-resolution computed tomography (CT) to more accurately evaluate the morphology and thickness of the bony septum. In future similar cases, incorporating CT imaging is recommended to enhance the reliability of anatomical conclusions.

From a comparative literature perspective, schwannomas are benign tumors arising from peripheral nerve sheaths that may involve any cranial nerve. Isolated hypoglossal nerve palsy constitutes a rare manifestation of schwannoma, potentially causing ipsilateral hemi atrophy, tongue deviation, fasciculations, and meningeal irritation-induced headaches. While one prior case report documented schwannoma induced isolated hypoglossal palsy (6), our case provides the first detailed description of the pathogenic role played by the unique anatomical relationship between the hypoglossal canal and jugular foramen. This discovery offers novel insights into understanding the diverse clinical presentations of skull base lesions. In clinical practice, such awareness enhances physician vigilance when encountering atypical cranial nerve symptoms, thereby reducing diagnostic oversights or errors.

## Conclusion

This case report provides a comprehensive documentation of the diagnostic and therapeutic management of a rare jugular foramen tumor causing isolated hypoglossal nerve injury. Through this case, we highlight the critical value of high-resolution imaging in diagnosing skull base lesions, with particular emphasis on the indispensable role of multi-sequence MRI in delineating the anatomical relationship between neural structures and pathological lesions. Furthermore, our experience demonstrates the necessity of multidisciplinary collaboration in determining treatment strategies for complex skull base pathologies, as well as the importance of selecting therapeutic approaches tailored to both tumor characteristics and individual anatomical variations. The atypical presentation in this case serves as an important reminder for clinicians to maintain a broad differential diagnosis when evaluating isolated cranial nerve palsies, necessitating thorough investigation and comprehensive assessment. Moving forward, accumulation of additional similar cases will be essential to further elucidate optimal diagnostic and treatment algorithms for such uncommon pathological entities.

## Data Availability

The raw data supporting the conclusions of this article will be made available by the authors, without undue reservation.

## References

[1] Castling B Hicks K . Traumatic isolated unilateral hypoglossal nerve palsy–case report and review of the literature. Br J Oral Maxillofac Surg. (1995) 33:171–3. doi: 10.1016/0266-4356(95)90292-9, PMID: 7654663

[2] Mes M Palczewski P Szczudlik P Łusakowska A Maj E Gawel M . Hypoglossal nerve palsy as an isolated syndrome of internal carotid artery dissection: A review of the literature and a case report. Neurol Neurochir Pol. (2018) 52:731–5. doi: 10.1016/j.pjnns.2018.06.006, PMID: 30082078

[3] Panholzer J Kellermair L Eggers C . Hypoglossal nerve palsy after SARS-CoV-2 vaccination - report of two cases. BMC Neurol. (2022) 22:416. doi: 10.1186/s12883-022-02929-2, PMID: 36352369 PMC9643981

[4] Weindling SM Goff RD Wood CP DeLone DR Hoxworth JM . Is hypoglossal nerve palsy caused by craniocervical junction degenerative disease an underrecognized entity? AJNR Am J Neuroradiol. (2016) 37:2138–43. doi: 10.3174/ajnr.A4885, PMID: 27538906 PMC7963795

[5] Kawaura R Ohnishi M . Unilateral isolated hypoglossal nerve palsy secondary to tonsillitis. Cureus. (2021) 13:e20291. doi: 10.7759/cureus.20291, PMID: 35028202 PMC8747992

[6] Öztop-Çakmak Ö Vanli-Yavuz E Aygün S Bastan B Egemen E Solaroğlu I . Isolated hypoglossal nerve palsy due to a jugular foramen schwannoma. Ideggyogy Sz. (2019) 72(7-8):282–4. doi: 10.18071/isz.72.0282, PMID: 31517462

[7] Guarnizo A Glikstein R Torres C . Imaging Features of isolated hypoglossal nerve palsy. J Neuroradiol. (2020) 47:136–50. doi: 10.1016/j.neurad.2019.04.006, PMID: 31034896

[8] Aftahy AK Groll M Barz M Bernhardt D Combs SE Meyer B . Surgical management of jugular foramen schwannomas. Cancers (Basel). (2021) 13(16):218. doi: 10.3390/cancers13164218, PMID: 34439372 PMC8393280

[9] Ribeiro FV Sousa MP Palavani LB Andreão FF Feitosa Filho HN Cardoso LJC . Gamma knife radiosurgery for patients with jugular foramen schwannomas: systematic review and meta-analysis. Neurosurg Rev. (2025) 48(1):243. doi: 10.1007/s10143-025-03396-2, PMID: 39960644

